# Challenges, Innovations, and Training in Airway Management During Cardiopulmonary Resuscitation: A Narrative Review

**DOI:** 10.7759/cureus.71686

**Published:** 2024-10-17

**Authors:** Nobuyasu Komasawa

**Affiliations:** 1 Community Medicine Education Promotion Office, Faculty of Medicine, Kagawa University, Miki, JPN

**Keywords:** airway management, cardiopulmonary resuscitation, simulation, supraglottic device, videolaryngoscope

## Abstract

Emergency airway management is a crucial procedure performed to secure the airway and ensure effective ventilation during respiratory distress or airway obstruction. In the context of cardiopulmonary resuscitation (CPR), this process is complicated by factors such as anatomical challenges, environmental conditions, and the urgency of the situation. Recent advancements in airway management devices, including videolaryngoscopes and supraglottic devices (SGDs), have proven beneficial in managing these challenges. SGDs, such as the laryngeal mask airway (LMA) and i-gel®, provide an effective alternative when tracheal intubation is difficult or when continuous chest compressions are required. Furthermore, simulation-based education plays a critical role in training healthcare providers to handle complex airway scenarios during CPR. This review discusses the challenges of tracheal intubation during CPR, the usefulness of various devices including videolaryngoscopes and gum-elastic bougies (GEB), and the role of simulation training in improving outcomes. Ensuring proficiency in airway management techniques through hands-on practice and advanced simulation is essential for improving both survival rates and neurological outcomes in emergency settings.

## Introduction and background

Emergency airway management plays a critical role in supporting a patient's breathing during instances of respiratory distress or airway obstruction [1,2]. This life-saving intervention is often one of the first steps in an emergency setting, aiming to quickly secure the airway and ensure proper ventilation and oxygenation throughout the resuscitation process [3,4]. Failure to establish an airway effectively and promptly can result in hypoxia, brain damage, or death, underscoring the complexity and importance of this procedure. When performing airway management, it is vital to assess the patient's consciousness, breathing status, and any potential injuries swiftly. The chosen technique for airway management depends on several factors, including the patient's condition, the clinical environment, and available resources [5,6]. Typically performed by trained paramedics or physicians, this procedure requires specialized training that often includes simulations and hands-on practice to prepare practitioners for high-stakes situations.

A "difficult airway" scenario refers to cases where a healthcare provider, such as an anesthesiologist, encounters difficulty in either mask ventilation or tracheal intubation or both [7,8]. These difficulties may stem from factors related to the patient's anatomy, the provider's experience, or situational challenges such as environmental conditions [9,10]. For instance, during cardiac arrest, managing the airway becomes even more complicated due to the urgency of the situation and the patient's rapidly deteriorating state. Cardiac arrest often occurs outside controlled environments such as emergency rooms or operating theaters, where equipment and trained personnel are readily available, making intubation more straightforward [11,12]. In less controlled settings, such as hospital wards, corridors, or disaster sites, limitations in space and resources require healthcare providers to be adaptable, frequently using portable tools and innovative techniques [13]. Intubation with a conventional laryngoscope in these situations is further complicated by the need for a precise alignment of the oral, pharyngeal, and laryngeal axes [14]. Additionally, factors such as vomit, blood, or foreign objects can obstruct the glottic view, making it harder to visualize, especially during chest compressions, which adds to the risk of intubation failure [15,16].

Performing intubation during chest compressions presents additional challenges, due to not only anatomical complexities but also environmental and time-sensitive factors. Even brief delays can significantly affect patient outcomes. Continuous chest compressions are crucial for maintaining circulation during cardiopulmonary resuscitation (CPR), yet they hinder both the visualization of the glottis and the successful passage of the tracheal tube through the vocal cords [17,18]. Other elements, such as inadequate lighting, patient positioning (e.g., prone or lateral), and restricted access to necessary tools, can also make airway management more difficult in these situations [19]. Consequently, airway management during CPR often involves multiple challenges that demand not only technical skills but also the ability to remain focused and adaptable under high pressure [20]. Additional variables, including the clinical environment's intensity, patient positioning, and limitations in available tools, further complicate airway interventions. In these scenarios, successful management requires strong technical skills alongside effective communication and teamwork within the resuscitation team [21,22].

Recent advancements in airway management tools, such as videolaryngoscopes and supraglottic devices (SGDs) such as the laryngeal mask airway (LMA), have demonstrated increased utility in emergency settings [23]. Videolaryngoscopes, for example, provide an indirect view of the glottis, improving visualization in difficult intubation cases. Meanwhile, SGDs offer an alternative means of securing the airway when tracheal intubation is not immediately possible [24]. These devices have shown promise in reducing the difficulty of intubation and minimizing interruptions during CPR. The current guidelines for CPR also recommend the use of these advanced airway management devices for their potential to improve patient outcomes. However, using such devices is not without challenges, as they may prolong interruptions in chest compressions, which can reduce blood flow and survival chances [25,26]. Therefore, it is crucial to weigh the benefits and risks of advanced airway devices in emergency situations carefully. On the other hand, if airway management can be accomplished without halting chest compressions, it may improve survival rates and neurological outcomes [27].

This review explores three key topics: (I) the challenges related to tracheal intubation during CPR and the role of tools such as indirect videolaryngoscopes and the gum-elastic bougie (GEB), (II) the effectiveness of various supraglottic devices during CPR, and (III) the significance of simulation-based training in mastering the use of these devices. Simulation-based education has become a fundamental aspect of training, allowing healthcare providers to refine their airway management techniques in a controlled, realistic setting. Such training not only enhances technical skills but also fosters better decision-making, teamwork, and stress management in actual emergency situations.

## Review

Problems with tracheal intubation during cardiopulmonary resuscitation (CPR) and the usefulness of indirect videolaryngoscopes and gum-elastic bougie

According to the 2020 guidelines, mastering bag-valve-mask (BVM) ventilation is a fundamental skill for all rescuers involved in securing the airway during chest compressions in CPR, as it ensures effective oxygen delivery. Additionally, rescuers trained in advanced cardiovascular life support (ACLS) should possess expertise in advanced airway management techniques, with an awareness of the possibility of inadequate ventilation [28]. The key benefit of tracheal intubation during CPR is that it allows uninterrupted chest compressions by securing the airway. Maintaining continuous chest compressions is vital for preserving cerebral and coronary perfusion. Properly securing the airway and providing appropriate ventilation also help enhance oxygenation, prevent hyperventilation (which can negatively impact cerebral and coronary blood flow), and reduce the risk of aspiration caused by gastric contents, potentially improving resuscitation outcomes [29,30]. However, tracheal intubation during chest compressions can be challenging and often leads to extended interruptions. The 2020 guidelines recommend that decisions regarding tracheal intubation should consider the potential interruptions to chest compressions, the patient's condition, and the rescuer's proficiency in airway management [28].

When tracheal intubation can be successfully performed without pausing chest compressions, it offers distinct advantages. What factors contribute to the difficulty of intubation during cardiopulmonary resuscitation (CPR) compressions? The use of a laryngoscope during compressions involves positioning the blade in the vallecula, and the movement of the head caused by compressions interferes with the alignment of the oral, pharyngeal, and laryngeal axes, making the visualization of the glottis more difficult [31]. Additionally, the synchronized movement of the glottis and tracheal tube with chest compressions complicates the passage of the tube through the vocal cords. To overcome these challenges, devices such as the Airway Scope® (AWS), Airtraq® (ATQ), and McGRATH® MAC have been designed to simplify intubation and enhance success rates during chest compressions [32,33]. Simulation studies have shown that AWS use during chest compressions significantly improves intubation success rates and shortens the time required for the procedure compared to traditional laryngoscopes [34,35]. The AWS features a blade with a tracheal tube attached, allowing the glottis, tube, and target marker to move in sync with the patient's chest during compressions. This synchronized movement ensures that the relative position between the glottis and the tube remains stable, simplifying intubation based on the screen view provided by the AWS.

In cases of traumatic cardiac arrest, it is essential to immobilize the cervical spine until a spinal injury can be ruled out. Laryngoscopes can exert significant force on the cervical spine, but indirect laryngoscopes such as the AWS can reduce cervical movement, making them particularly useful for intubation during CPR when cervical protection is required [18]. Although these indirect laryngoscopes have shown promise in both clinical reports and simulation studies, challenges remain. For example, chest compressions can cause the monitor's view to become obscured by condensation, vomit, or other bodily fluids, making it difficult to maintain a clear visual field. These issues underscore the need for further refinement and development.

The gum-elastic bougie (GEB) is another widely used tool in airway management, and many clinical guidelines recommend its early use in difficult intubation cases [36,37]. Numerous studies have evaluated its efficacy in managing challenging adult airways, particularly when laryngoscopy proves difficult [37,38]. The GEB is an important device for securing the airway, and several guidelines advocate for its early use in cases of difficult intubation. Research involving adult patients suggests that the GEB can significantly improve intubation success rates. In simulation studies, the success rate of intubation during chest compressions improved markedly with the use of the GEB [39]. Furthermore, tracheal intubation success was significantly higher when using the GEB in the presence of vomitus or hematemesis [40]. This may be due to the tactile feedback, such as the "clicks" and "hold-up" sensation, that even inexperienced practitioners can feel when using the bougie, facilitating more precise intubation [41]. Future investigations should explore the combination of videolaryngoscopes and the GEB to further enhance airway management during chest compressions.

Airway management heroes: The role and evolution of SGDs in resuscitation

Supraglottic devices (SGDs), such as the laryngeal mask airway (LMA) and i-gel®, are specifically designed to secure the airway and assist with ventilation [42]. Unlike tracheal intubation, SGDs do not require a direct visualization of the vocal cords, which allows for easier insertion without disrupting chest compressions. Moreover, the training required for both inserting these devices and retaining proficiency is relatively straightforward. Given that SGDs can provide airway security when other methods, such as mask ventilation or tracheal intubation, prove difficult, their early use is often recommended in emergency scenarios. SGDs are typically considered more reliable than face mask ventilation techniques. While they do not eliminate the risk of aspiration entirely, the occurrence is generally lower compared to bag-valve-mask (BVM) ventilation. Furthermore, inserting an SGD is simpler to master compared to tracheal intubation and can be effectively utilized even in situations involving unstable cervical spine injuries or varied patient positions. According to the 2020 guidelines, trained responders can opt for SGDs as an alternative to BVM or tracheal intubation during cardiac arrest situations [28].

Recent advancements in the anatomical design of SGDs have improved their effectiveness during chest compressions. Studies using simulation with junior residents have demonstrated that devices such as the LMA Supreme® or air-Q® significantly increase the rate of first ventilation success during chest compressions when compared to traditional SGDs [6,43]. This enhancement is likely due to the anatomical-shaped SGDs to create a strong airway seal and the inclusion of a gastric tube port, which helps to release air from the stomach. The American Society of Anesthesiologists (ASA) introduced its Difficult Airway Algorithm (DAA) in 1993, and it has been updated several times since. The adoption of the LMA prompted a revision of the algorithm in 2003, recommending its early use. Initially developed as minimally invasive tools, SGDs have evolved into critical instruments for managing upper airway obstructions, and they are now widely recognized as indispensable in difficult airway management, especially among anesthesiologists.

In the context of cardiopulmonary resuscitation (CPR), several factors, including the stressful environment, patient positioning, limitations of devices, and the need for uninterrupted chest compressions, can create what is referred to as a "difficult airway." Although the ASA Difficult Airway Management (DAM) guidelines recognize the value of SGDs, the 2020 guidelines also emphasize their potential role in addressing difficult airways during emergency scenarios [28]. A range of LMAs and other SGDs has been developed in recent years, and further research into their effectiveness during both difficult airway management and CPR is expected. Choosing the most appropriate SGD depends on factors such as the level of training required, airway protection, ventilation efficiency, and challenges associated with patient transport. Some SGDs, such as the Fastrack®, aura-i®, air-Q®, and i-gel®, are classified as intubating supraglottic devices (ISGDs) because they enable tracheal intubation following successful ventilation [44]. Since the primary objective of airway management during resuscitation is effective oxygenation, SGDs are valuable for their ability to rapidly and reliably establish ventilation. Additionally, ISGDs allow for secure tracheal intubation after proper ventilation has been established [45,46].

SGDs have demonstrated their effectiveness not only in resolving upper airway obstruction, as outlined in the ASA-DAA for anesthesiologists, but also as essential emergency airway management tools for non-anesthesiologists, as noted in the 2020 American Heart Association (AHA) guidelines. This makes them versatile devices, suitable for managing sudden ventilation difficulties or for use in cardiac arrest cases. Certainly, further evaluation is needed for rapid size determination or application in vomitus cases. Because no single technique guarantees a complete solution to airway management challenges, it is critical to anticipate potential difficulties and be prepared to adapt swiftly.

Training and simulation for airway management during CPR

The 2020 guidelines emphasize the importance of regular training for rescuers to maintain competence in advanced airway management techniques. In case the initial airway strategy fails, a backup plan should always be ready. For example, rescuers should be proficient in bag-valve-mask (BVM) ventilation, which includes the use of oral and nasal airways, as well as two-person ventilation methods. Familiarity with a range of airway management devices, such as supraglottic devices (SGDs), for example, the laryngeal mask airway (LMA), and various videolaryngoscopes for intubation, should be developed through consistent practice to ensure effective airway management during emergencies. Training for medical procedures typically falls into two categories: on-the-job training and off-the-job training. On-the-job training provides hands-on experience during clinical practice, while off-the-job training involves learning from textbooks and participating in simulation-based CPR training using mannequins. In emergency airway situations, it is often challenging to replicate the controlled environment of a scheduled operating room. Moreover, as the 2020 guidelines indicate, mastering a variety of airway management tools is crucial, including laryngoscopy and SGDs. One of the main challenges is identifying which techniques should be prioritized during clinical training, particularly in the early stages of medical education. Additionally, training in critical situations on the job poses ethical concerns, as patient safety must always remain the top priority. Therefore, simulation-based education plays a vital role in supplementing clinical experience.

To overcome the limitations of on-the-job training for airway management, simulation-based training is an effective method. Globally implemented CPR training programs, such as basic life support (BLS) and advanced life support (ALS), have demonstrated the value of simulation by providing healthcare providers with practical experience in techniques that are difficult to acquire solely through clinical practice. Fields such as anesthesiology and emergency medicine adopted simulation-based training, including ALS, earlier than many other specialties [47]. In the American Heart Association (AHA) Advanced Life Support (ALS) provider course, which follows the 2020 guidelines, tracheal intubation is not heavily focused on due to the complexity of mastering this skill in short-term training [7]. Instead, the course prioritizes skills such as inserting oral and nasal airways, performing oral suction, administering oxygen, and performing two-person BVM ventilation. The guidelines also emphasize that personnel performing intubation during resuscitation should engage in continuous practice and retraining. Thus, in addition to short-term courses such as the two-day ALS provider course, ongoing simulation-based training is essential for improving proficiency in advanced airway management, including the use of SGDs and intubation techniques [48].

Encountering a difficult airway unexpectedly not only challenges the clinician's technical abilities but also tests the crisis management skills of the entire medical team, including doctors, nurses, and other healthcare professionals. To simulate real-life scenarios across various clinical settings, it is crucial to establish a cooperative team system. This involves not only doctors but also nurses, respiratory therapists, and clinical engineers. Effective teamwork requires strong communication and situational awareness, so simulation training should focus on improving both technical and nontechnical skills to promote successful collaboration in crisis situations [49]. Training with real healthcare teams in a hospital environment enhances the mutual understanding of roles and responsibilities, improves communication among clinicians and support staff, and fosters discussions on patient safety across departments. The 2020 AHA guidelines stress that healthcare providers should not rely on a single airway management technique but instead be skilled in multiple backup methods. Since airway emergencies occur in diverse clinical contexts, mastering various techniques, including the use of SGDs, increases the likelihood of successful airway management in critical situations [50]. Team-based training in a wide range of airway management techniques is invaluable for all healthcare professionals involved in both emergency and routine care, ensuring preparedness for the wide array of airway and ventilation challenges that can arise.

## Conclusions

Emergency airway management is a life-saving procedure that must be performed rapidly and accurately in cases of respiratory distress or airway obstruction. However, securing the airway during cardiopulmonary resuscitation (CPR) presents additional challenges due to anatomical and environmental factors. Recent advances in airway management devices, such as videolaryngoscopes and supraglottic devices (SGDs), have proven useful in these difficult situations. SGDs, in particular, are recommended for maintaining the airway without interrupting chest compressions. Simulation-based education is also essential, allowing healthcare providers to practice in realistic settings, enhancing both technical skills and teamwork.
